# Elevated Bile Acids in Newborns with Biliary Atresia (BA)

**DOI:** 10.1371/journal.pone.0049270

**Published:** 2012-11-14

**Authors:** Kejun Zhou, Na Lin, Yongtao Xiao, Yang Wang, Jie Wen, Gang-Ming Zou, XueFan Gu, Wei Cai

**Affiliations:** 1 Department of Pediatric Surgery, Xin Hua Hospital, School of Medicine, Shanghai Jiao Tong University, Shanghai, China; 2 Shanghai Key Laboratory of Pediatric Gastroenterology and Nutrition, Shanghai, China; 3 Shanghai Institute of Pediatric Research, Shanghai, China; 4 Department of Pediatrics, Xinhua Hospital, School of Medicine, Shanghai Jiao Tong University, Shanghai, China; Ospedale Pediatrico Bambino Gesu', Italy

## Abstract

Biliary Atresia (BA), a result from inflammatory destruction of the intrahepatic and extrahepatic bile ducts, is a severe hepatobiliary disorder unique to infancy. Early diagnosis and Kasai operation greatly improve the outcome of BA patients, which encourages the development of early screening methods. Using HPLC coupled tandem mass spectrometry, we detected primary bile acids content in dried blood spots obtained from 8 BA infants, 17 neonatal jaundice and 292 comparison infants at 3–4 days of life. Taurocholate (TC) was significantly elevated in biliary atresia infants (0.98±0.62 µmol/L) compared to neonatal jaundice (0.47±0.30 µmol/L) and comparison infants (0.43±0.40 µmol/L), with p = 0.0231 and p = 0.0016 respectively. The area under receiver operating characteristic (ROC) curve for TC to discriminate BA and comparison infants was 0.82 (95% confidence interval: 0.72–0.92). A cutoff of 0.63 µmol/L produced a sensitivity of 79.1% and specificity of 62.5%. The concentrations of total bile acids were also raised significantly in BA compared to comparison infants (6.62±3.89 µmol/L vs 3.81±3.06 µmol/L, p = 0.0162), with the area under ROC curve of 0.75 (95% confidence interval: 0.61–0.89). No significant difference was found between the bile acids of neonatal jaundice and that of comparison infants. The early increase of bile acids indicates the presentation of BA in the immediate newborn period and the possibility of TC as newborn screening marker.

## Introduction

Biliary atresia (BA) is the most significant life-threatening hepatobiliary disorder in children which results from destructive inflammatory process of both intrahepatic and extrahepatic bile ducts [1], [2]. If untreated, progressive liver cirrhosis leads to death by 2 years of age. Clinical features of BA include jaundice, pale stools and dark urine, shown at or soon after birth with normal birth weights. The etiology of BA is largely unknown. Diagnosis of BA is still difficult and operative cholangiography to visualize the biliary tract is still the golden standard for the diagnosis [2]. Kasai portoenterostomy is the first choice of treatment and good results depend on early diagnosis and operation [3], [4], [5]. Since the use of the infant stool color card to screen BA in Taiwan, BA patients have been diagnosed earlier and the outcomes have been improved remarkably [6]. However this screening method is greatly affected by parents' subjectivity. Because of the importance of early diagnosis and treatment, a great deal of efforts have been made to develop early detection methods for BA. For instance, conjugated bilirubin in plasma measured at 6–10 days is a reliable marker for neonatal liver disease [7]. Furthermore, elevated direct/conjugated bilirubin levels were found shortly after birth (within 24–72 hours) in BA infants [8]. Unfortunately, conjugated bilirubin cannot be detected in dried blood spots because of its light sensitivity.

Bile acids, as other important makers related with liver injury and cholestasis , are widely investigated to screen or discriminate cholestasis diseases, including BA [9], intrahepatic cholestasis of pregnancy [10] and bile acid synthesis defects [11]. With the widespread application of tandem mass spectrometry, this technology was used to detect bile acids in dried blood spots [12], [13], [14]. To evaluate if bile acids can be used as early screening marker for BA, we detected the six primary bile acids in dried blood spots of BA at 3–4 days after birth.

## Methods

### BA, neonatal jaundice, and comparison group

BA and neonatal jaundice cases were identified from Xin Hua Hospital, Shanghai, China. BA cases were diagnosed by cholangiography. All the neonatal jaundice infants shown jaundice within 4 days after birth. Dried blood spots originally collected for screening of phenylketonuria were used for this study. All the dried blood spots were stored at −20°C after phenylketonuria screening. Anonymized screening cards of the same month were randomly selected to provide a comparison group of each BA card. Ethical approval was obtained from the ethics committee of Xin Hua Hospital. Written informed consent was obtained from parents of all BA and neonatal jaundice infants and oral consent was obtained from parents of all comparison infants. Serum total and direct bilirubin when these patients were hospitalized were listed in Table S1.

### Calibrators and Sample analysis

Stock solutions of 500 µmol/L of primary bile acids (cholic acid : CA, glycocholate : GC, taurocholate : TC, chenodeoxycholate : CDC, glycochenodeoxycholate : GCDC, taurochenodeoxycholate : TCDC) were used to prepare calibrators. Whole blood with a hematocrit of 55% active carbon desalted serum was spiked with proper amount of stock solution to achieve final concentrations of 20, 5.0, 1.0, 0.5, 0.1, 0.05 and 0 µmol/L bile acids. Twenty-five micro liter of spiked whole blood was used for the preparation of blood spot standards on filter paper. Calibrators were stored at −20°C.

Concentrations of the six primary bile acids were detected in dried blood spots with an API 4000 MS coupled with a HPLC system according to the method described by Janzen et al with small modifications[13]. Three millimeter discs were punched and incubated in 150 µl methanol with 0.5 µmol/L cholic-2,2,4,4-d 4 acid (d 4 –CA, internal standard) for 30 min. Ten micro liter of supernatant was injected and separation was achieved using a Luna C18 column of 50×2.0 mm, 3 µmol/L, which was protected by a C18 4×2.0 mm Precolumn from Phenomex (Aschaffenburg, Germany). Data acquisition was performed using Analyst 1.5 software from Applied Biosystems. Analytical settings and limit of quantification for the six primary bile acids were listed in Table S2. Total bile acid concentration was calculated from sum of the six bile acids [15].

### Statistical analysis

The data of bile acids were tested for normal distribution using the Shapiro-Wilk test before calculation of differences. Normally distributed data was analyzed using one-way ANOVA followed by a Post Hoc Dunnett Test. Non-normally distributed data was analyzed using the Wilcox test or the Kruskal test. A p-value of <0.05 was considered statistically significant. Receiver operating characteristic (ROC) curves were plotted to compare the screening ability of these bile acids. All the analysis was carried out on the freely available software R with corresponding packages. ROC curves and box-plots of these bile acids were drawn by SPSS version 19.0.

## Results

Dried blood spots from 292 comparison infants, 17 neonatal jaundice and 8 biliary atresia infants were enrolled in this study. Six primary bile acids (CA, GC, TC, CDC, GCDC, and TCDC) were measured in the dried blood spots with API 4000 tandem MS. Figure S1 presents a representative LC-MS/MS chromatogram of bile acids in dried blood spots from BA. The concentrations of conjugated primary bile acids were much higher than the un-conjugated ones. The CA and CDC concentrations in most cases were under the quantification limits. Hence, data of these two bile acids was not analyzed in the following study. The data of conjugated primary bile acids in comparison group was tested for normal distribution using the Shapiro-Wilk Test initially. Because none of the data was normally distributed (data not shown), the Wilcox test was used to analyze the differences among BA, neonatal jaundice and comparison infants.

**Figure 1:**
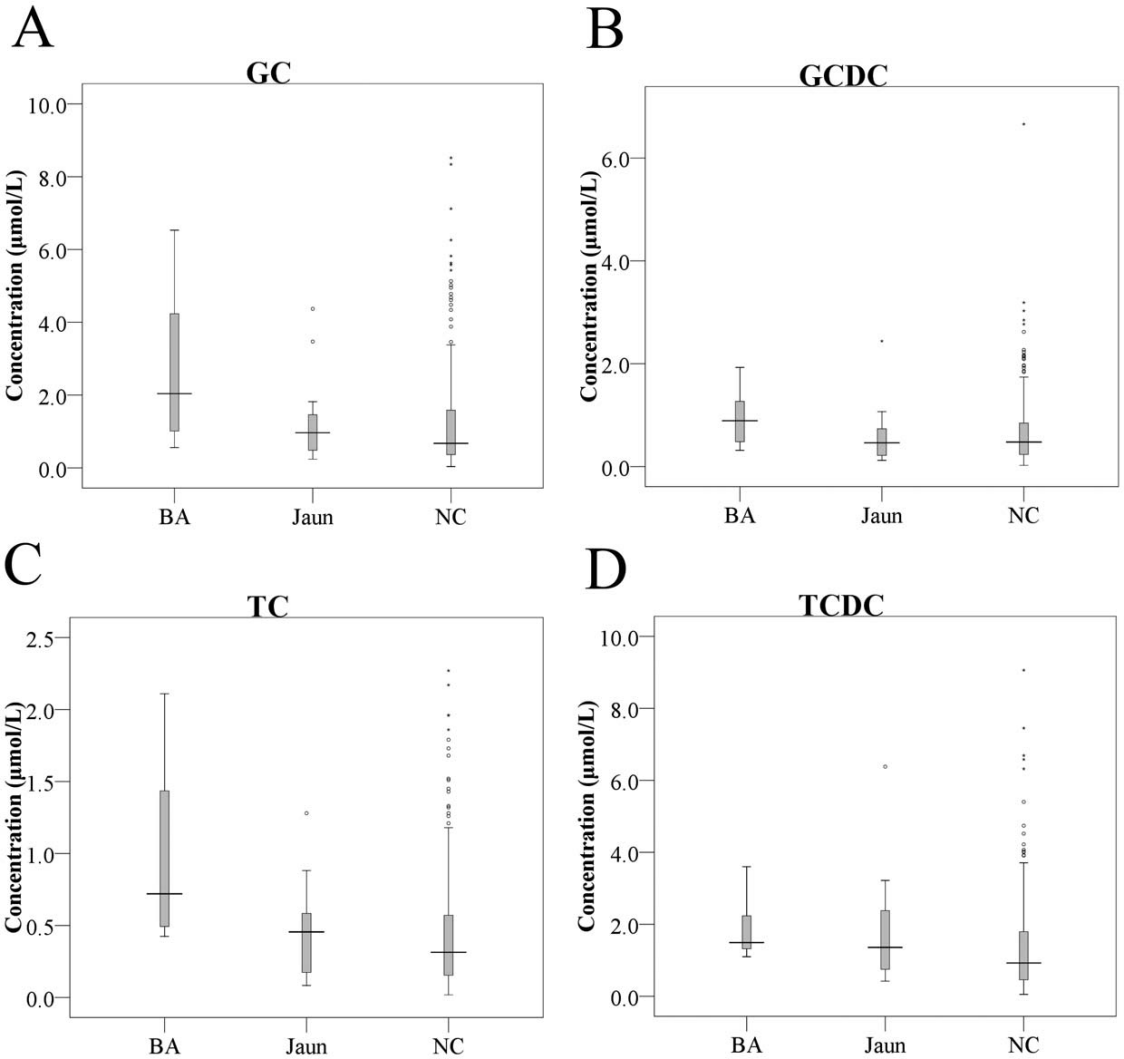
Boxplots of GC (A), GCDC (B), TC (C) and TCDC (D) in dried blood spots of biliary atresia (n = 8), neonatal jaundice (n = 17) and comparison infants (n = 292). The concentrations of GC, GCDC, TC, and TCDC in BA were significantly higher than those of comparison infants (p<0.05). TC was also significantly elevated in BA compared to neonatal jaundice. No significant difference was observed between neonatal jaundice and comparison infants. BA: biliary atresia infants, Jaun: neonatal jaundice infants, NC: comparison infants.

**Figure 2:**
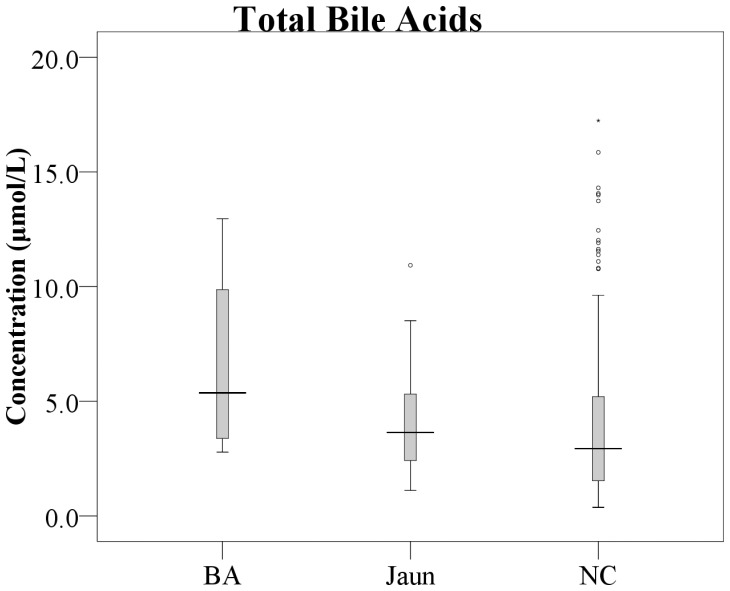
Boxplot of total bile acids in dried blood spots of biliary atresia (n = 8), neonatal jaundice (n = 17) and comparison infants (n = 292). The level of total bile acids in BA was raised significantly compared to comparison infants, with p = 0.0162. BA: biliary atresia infants, Jaun: neonatal jaundice infants, NC: comparison infants.

Concentrations of the four conjugated bile acids and total bile acids were significantly higher in BA than comparison infants (table 1). However, great overlaps existed between the two groups (Figure 1 and 2). TC was most significantly elevated in BA infants (0.98±0.62 µmol/L) compared to neonatal jaundice (0.47±0.30 µmol/L) and comparison infants (0.43±0.40 µmol/L), with p = 0.0231 and p = 0.0016 respectively. The means of GC and GCDC were higher in BA than in neonatal jaundice infants with marginal significance (GC: 2.71±2.26 µmol/L vs 1.31±1.11 µmol/L, p = 0.0664; GCDC: 0.94±0.55 µmol/L vs 0.63±0.54 µmol/L, p = 0.0967). No significant difference was found between neonatal jaundice and the comparison infants except a minor increase of TCDC (1.82±1.47 µmol/L vs 1.34±1.29 µmol/L, p = 0.0591).

**Table 1:** Bile acids concentrations (mean±SD) in dried blood spots of BA, neonatal jaundice and comparison infants.

Variables	Comparison group (n = 292)	Jaundice group (n = 17)	BA group (n = 8)	P value		
				Comparison vs Jaundice	Comparison vs BA	Jaundice vs BA
GC (µmol/L)	1.24±1.40	1.31±1.11	2.71±2.26	0.2551	***0.0139***	0.0664
GCDC (µmol/L)	0.65±0.66	0.63±0.54	0.94±0.55	0.7747	***0.0487***	0.0967
TC (µmol/L)	0.43±0.40	0.47±0.30	0.98±0.62	0.2409	***0.0016***	***0.0231***
TCDC (µmol/L)	1.34±1.29	1.82±1.47	1.85±0.82	0.05906	***0.0304***	0.5216
Total bile Acids (µmol/L)	3.81±3.06	4.36±2.64	6.62±3.89	0.2787	***0.0162***	0.1299

**Figure 3:**
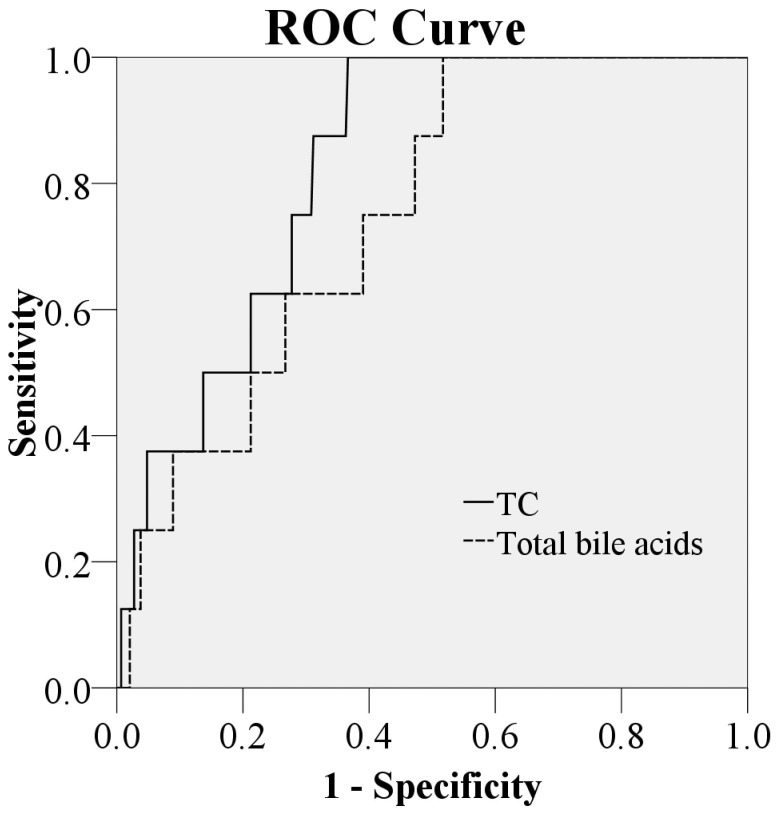
Receiver operating charactristic curves to discriminate BA and comparison infants for concentrations of TC and total bile acids. The area under the curve was 0.82 (95% confidence interval: 0.72–0.92) for TC and 0.75 (95% confidence interval: 0.61–0.89) respectively, with p = 0.01976.

Figure 3 shows receiver operating characteristic curves for TC and total bile acids to discriminate BA and comparison infants. The area under the ROC curve for TC was 0.82 ( 95% confidence interval: 0.72–0.92) which was significantly larger than total bile acids (with the area under ROC curve of 0.75, 95% confidence interval: 0.61–0.89) with p = 0.01976. Besides, the sensitivity and specificity values for various TC cutoff levels were calculated (Table 2). A cutoff of 0.63 µmol/L produced a sensitivity of 79.1%, specificity of 62.5%.

**Table 2:** Sensitivity and specificity of TC concentration in prediction of BA.

Cutoff	% Sensitivity (95% CI)	% Specificity (95% CI)
0.7985	86.6(82.5–90.1)	50(12.5–87.5)
**0.63**	**79.1(74.3–83.2)**	**62.5(25–87.5)**
0.5	72.3(67.5–77.4)	75(37.5–100)
0.4735	68.8(63.7–74.0)	87.5(62.5–100)
0.4225	63.4(58.2–68.5)	100(100–100)

To analyze the difference among different storage time, different gestational weeks and different genders, the Kruskal test was also carried out. The conjugated primary bile acids show no difference between different storage time, gestation weeks and genders (data not shown).

## Discussion

Biliary atresia is the most common cause of neonatal cholestasis and 40–50% of liver transplantation in children is caused by this disease [16]. Early intervention of Kasai portoenterostomy is very important for BA patients. When the surgery is performed before the age of 60 days, the success rate of establishing biliary drainage is higher than those performed later [17] and the need for liver transplantations in infancy and childhood is greatly reduced [5].

In this study, we evaluated the six primary bile acids in dried blood spots of BA, neonatal jaundice and comparison infants obtained 3–4 days after birth. All the conjugated primary bile acids have already increased in BA newborns at 3–4 days of life which echoes the report that all these bile acids were elevated significantly in BA infants 7–10 days after birth [15]. The results indicated that BA was present in the immediate newborn period or late pregnancy. This is also supported by a case report by Toubi et al., where a BA infants with disappeared gallbladder at age of 11 days was used to have normal gallbladder at the gestational age of 24 weeks [18]. In our study, the increase of TC and GC was higher than TCDC and GCDC in early life of BA, while Mills et al. found that the later 2 bile acids increased more significant in cholestatic children at age of 3 weeks to 8 month [19]. These findings may indicate that the liver damage has already begun but is still mild in BA infants within 3–4 days of age as greater rise of TCDC and GCDC meant severe liver damage [20]. Furthermore, TC was found positively correlated with MCP-1, an HSC-responsive chemokine, in cholestatic rats and in cystic fibrosis liver disease [21]. The early elevation of TC may indicate the early occurrence of fibrosis in BA patients.

According to previous research, two of the most promising screening methods for BA yet are early measurement of serum conjugated bilirubin and usage of stool color cards. In the test of serum conjugated bilirubin at 6–10 days after birth, the sensitivity and specificity reach 100% and 99.6% respectively [7]. However, due to the light sensitivity of bilirubin, it is unsuitable to detect in dried blood spots, which are easy to obtain and deliver for newborn screening. Another method, the stool color card is widely used in Taiwan to screen BA patients. The sensitivity of this method was 97.1% in 2005 [22]. However the onset of pale stool in a large amount of cases usually occurs later than 30 days of age, which may delay the diagnosis and affect the outcome of treatment. Measurement of TC in dried blood spots by LC-MS/MS may be suitable for newborn screening due to the stability in dried blood spots and relatively simple detection technique, although the sensitivity and specificity are not as good as serum conjugated bilirubin and the stool color card. Furthermore, considering the effect of diurnal variations on the level of bile acids in blood [23], the detecting accuracy may increase if all blood spots were taken forenoon. After all, the elevated concentrations of TC and other bile acids in dried blood spots from BA infants have already indicted the potential screening strategy for the future study.

Interestingly, no significant difference of bile acids was observed between neonatal jaundice and comparison infants. This result was understandable. It is well known that genetic and environmental factors influence neonatal jaundice, such as ABO incompatibility, breastfeeding and the method of delivery [24]. The infants with neonatal jaundice enrolled in this study have unconjugated hyperbilirubinemia which are unrelated with hepatobiliary disease.

The main shortage of this study is the small sample size of BA. Because of the scarcity of the disease, insufficient therapeutic effects and low levels of cooperation among pediatric hospitals in China, it is hard to obtain screening dried blood spots from BA patients. Furthermore, the detection methods need to be improved, which include but are not limited to the introduction of UPLC, more sensitive MS and proper sample pretreatment methods.

In conclusion, bile acids were elevated in BA infants at 3–4 days of age indicates the immediate occurrence of the disease at birth. The measurement of bile acids, especially TC, in dried blood spots from newborns could be the potential screening strategy for early detection of BA.

## Supporting Information

Table S1 Serum total bilirubin and direct bilirubin in patients with biliary atresia and neonatal jaundice.
(DOCX)

Table S2 Analytical settings and limit of quantification of the primary bile acids in dried blood spots.
(DOCX)

Figure S1 A representative chromatogram of bile acids in a dried blood spot from a biliary atresia infant with 0.5 µM d4-CA. The concentrations of conjugated primary bile acids were much higher than unconjugated ones. Unconjugated bile acids, CA and CDC, were under the quantification limits.
(DOCX)
