# Supplementary material for: Elevated Bile Acids in Newborns with Biliary Atresia (BA)
Source: PLoS One. 2012 Nov 14;7(11):e49270. doi: 10.1371/journal.pone.0049270 (PMC3498146; doi:10.1371/journal.pone.0049270)
Supplement: Table S1 — Serum total bilirubin and direct bilirubin in patients with biliary atresia and neonatal jaundice. (DOCX) [file pone.0049270.s001.docx]

Table S1: Serum total bilirubin and direct bilirubin in patients with biliary atresia and neonatal jaundice.

| **Patients** | **Gender** | **AGE (day)** | **TB (μmol/L)** | **DB (μmol/L)** |
| --- | --- | --- | --- | --- |
| BA01 | Female | 70 | 154.1 | 117.9 |
| BA02 | Male | 43 | 215.8 | 105.9 |
| BA03 | Female | 65 | 125.7 | 90.5 |
| BA04 | Female | 31 | 102 | 54.6 |
| BA05 | Male | 70 | 125 | 100 |
| BA06 | Female | 26 | 139 | 102 |
| BA07 | Male | 48 | 162.3 | 95.8 |
| BA08 | Male | 52 | 220.8 | 169.8 |
| Jaun01 | Male | 11 | 159.2 | 12.4 |
| Jaun02 | Female | 20 | 203.7 | 15.4 |
| Jaun03 | Female | 33 | 162.1 | 14.7 |
| Jaun04 | Female | 34 | 271.5 | 12.6 |
| Jaun05 | Male | 26 | 184.5 | 18.6 |
| Jaun06 | Female | 23 | 192.7 | 15.8 |
| Jaun07 | Male | 38 | 123.6 | 13.9 |
| Jaun08 | Male | 7 | 224.1 | 20.1 |
| Jaun09 | Male | 17 | 206.4 | 21.2 |
| Jaun10 | Male | 4 | 180.9 | 11.8 |
| Jaun11 | Female | 32 | 187.6 | 17.1 |
| Jaun12 | Female | 19 | 163.9 | 11.3 |
| Jaun13 | Female | 30 | 209.9 | 13.4 |
| Jaun14 | Male | 18 | 162.3 | 14.3 |
| Jaun15 | Male | 13 | 187.7 | 12.6 |
| Jaun16 | Female | 14 | 214.7 | 9.6 |
| Jaun17 | Female | 15 | 195.1 | 12.4 |

BA, biliary atresia; Jaun, neonatal jaundice; TB, toatal bilirubin; DB, direct bilirubin.
