# Supplementary material for: Elevated Bile Acids in Newborns with Biliary Atresia (BA)
Source: PLoS One. 2012 Nov 14;7(11):e49270. doi: 10.1371/journal.pone.0049270 (PMC3498146; doi:10.1371/journal.pone.0049270)
Supplement: Table S2 — Analytical settings and limit of quantification of the primary bile acids in dried blood spots. (DOCX) [file pone.0049270.s002.docx]

Table S2: Analytical settings and limit of quantification of the primary bile acids in dried blood spots.

|  | Parent [m/z] | Daughter [m/z] | DP | CE | CXP | RT [min] | LOQ [μM] |
| --- | --- | --- | --- | --- | --- | --- | --- |
| d4-CA | 411,3 | 347,3 | -125 | -48 | -6 | 5.02 |  |
| CA | 407,2 | 343,3 | -145 | -44 | -5 | 5.03 | 0.05 |
| GC | 464,2 | 73,9 | -140 | -68 | -11 | 2.63 | 0.05 |
| TC | 514,2 | 80,0 | -165 | -115 | -11 | 2.48 | 0.05 |
| CDC | 391,2 | 391,2 | -140 | -44 | -8 | 7.30 | 0.1 |
| GCDC | 448,2 | 73,9 | -105 | -50 | -10 | 4.01 | 0.1 |
| TCDC | 498,2 | 79,9 | -195 | -110 | -1 | 3.82 | 0.1 |

DP, declustering potential; CE, collision energy; CXP, collision cell exit potential; RT, retention time; LOQ, limit of quantification; CA, cholic acid ; GC, glycocholate; TC, taurocholate; CDC, chenodeoxycholate; GCDC, glycochenodeoxycholate; TCDC, taurochenodeoxycholate.
