# Supplementary material for: Elevated Bile Acids in Newborns with Biliary Atresia (BA)
Source: PLoS One. 2012 Nov 14;7(11):e49270. doi: 10.1371/journal.pone.0049270 (PMC3498146; doi:10.1371/journal.pone.0049270)

Figure S1: A representative chromatogram of bile acids in a dried blood spot from a biliary atresia infant with 0.5 μM d4-CA. The concentrations of conjugated primary bile acids were much higher than unconjugated ones. Unconjugated bile acids, CA and CDC, were under the quantification limits. TC, taurocholate; GC, glycocholate; TCDC, taurochenodeoxycholate; GCDC, glycochenodeoxycholate; d4-CA, cholic-2,2,4,4-d4 acid.


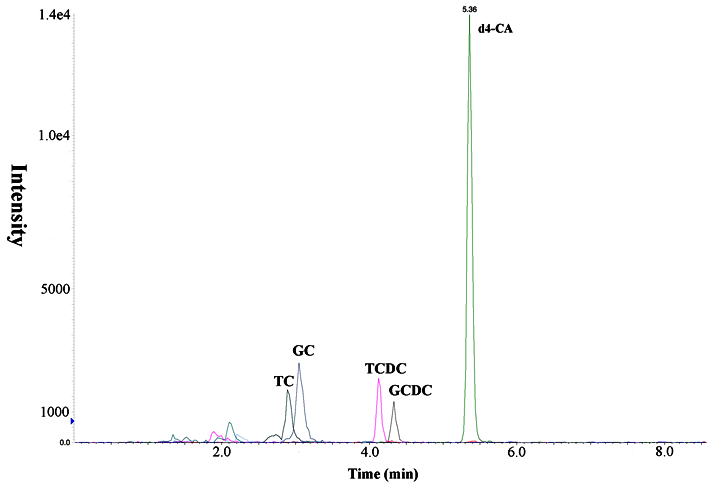

Supplement: Figure S1 — A representative chromatogram of bile acids in a dried blood spot from a biliary atresia infant with 0.5 µM d4-CA. The concentrations of conjugated primary bile acids were much higher than unconjugated ones. Unconjugated bile acids, CA and CDC, were under the quantification limits. (DOCX) [file pone.0049270.s003.docx]
